# Comparison between the BACTEC MGIT 960 system and the agar proportion method for susceptibility testing of multidrug resistant tuberculosis strains in a high burden setting of South Africa

**DOI:** 10.1186/1471-2334-12-369

**Published:** 2012-12-22

**Authors:** Halima M Said, Marleen M Kock, Nazir A Ismail, Kamaldeen Baba, Shaheed V Omar, Ayman G Osman, Anwar A Hoosen, Marthie M Ehlers

**Affiliations:** 1 Department of Medical Microbiology, Faculty of Health Science, University of Pretoria, Private bag X323, arcadia, Pretoria, 0007, South Africa; 2 National Health Laboratory Service, Tshwane Academic Division, Pretoria, South Africa

**Keywords:** BACTEC MGIT 960, MDR-TB, TB, XDR-TB

## Abstract

**Background:**

The increasing problem of multi-drug-resistant (MDR) tuberculosis (TB) [ie resistant to at least isoniazid (INH) and rifampicin (RIF)] is becoming a global problem. Successful treatment outcome for MDR-TB depends on reliable and accurate drug susceptibility testing of first-line and second-line anti-TB drugs.

**Method:**

Consecutive *M. tuberculosis* isolates identified as MDR-TB during August 2007 to January 2008 using the BACTEC MGIT 960 systems and the agar proportion method were included in this study. Susceptibility testing of MDR-TB isolates against ethambutol (EMB) and streptomycin (STR) as well as two second-line anti-TB drugs, kanamycin (KAN) and ofloxacin (OFX) was performed using the BACTEC MGIT 960 systems at a routine diagnostic laboratory. The results were compared to those obtained by the agar proportion method.

**Result:**

The agreement between the BACTEC MGIT 960 system and the agar proportion method was 44% for EMB, 61% for STR and 89% for both KAN and OFX. The sensitivity and specificity of the BACTEC MGIT 960 system using the agar proportion method as a gold standard was 92% and 37% for EMB, 95% and 37% for STR, 27% and 97% for KAN and 84% and 90% for OFX, respectively.

**Conclusions:**

The BACTEC MGIT 960 system showed acceptable sensitivity for EMB, STR, and OFX; however, the BACTEC MGIT 960 system was less specific for EMB and STR and demonstrated a low sensitivity for KAN. The lower agreement found between the two methods suggests the unreliability of the BACTEC MGIT 960 system for the drugs tested. The reasons for the lower agreement between the two methods need to be investigated and further studies are needed in this setting to confirm the study finding.

## Background

Drug-resistance remains a serious threat to tuberculosis (TB) control programmes worldwide. South Africa is one of the high-burden multi-drug-resistant (MDR) TB [ie resistant to at least isoniazid (INH) and rifampicin (RIF)] countries in the world [1]. The emergence of extensively drug-resistant (XDR) TB [ie MDR-TB with additional resistance to any fluoroquinolone (FLQ) and to at least one of the three injectable second-line drugs: amikacin (AMK), kanamycin (KAN) and/or capreomycin (CAP)] is worsening the drug-resistance problem [1,2]. The World Health Organization (WHO) estimates that 10.5% of MDR-TB cases in South Africa are XDR-TB [1].

Standardised, rapid and accurate drug susceptibility testing (DST) methods for first-line and second-line anti-TB drugs is important to determine an effective treatment regimen and to decrease transmission. Various susceptibility testing methods are currently available especially for first-line anti-TB drugs [3,4]. However, the accuracy of these methods are reported to vary according to the anti-TB drug being tested [5]. Drug susceptibility testing for rifampicin (RIF) and isoniazid (INH) is most accurate, but less reliable and reproducible for streptomycin (STR), ethambutol (EMB) and pyrazinamide (PZA) [4-6]. Inconsistent results are a common occurrence [7]. This is particularly true in the case of EMB resistance as the diagnostic breakpoint (5 to 7.5 μg/ml) is close to the Minimal Inhibitory Concentration (MIC) of EMB and true resistance may therefore be missed [7].

Second-line DST is less standardised and not as simple as DST for first-line ant-TB drugs [5]. This is mainly due to *in-vitro* drug stability, varying drug potency and the critical concentration defining resistance is often close to the MIC [4,5]. These factors increase the probability for misclassification of susceptibility or resistance and leading to poor reproducibility of DST results [4,5].

Therefore, it is recommended to determine *in vitro* criteria, which could be used to predict clinical resistance and susceptibility with acceptable accuracy, by testing representative number of clinical isolates [4,5]. Although for several years the recommended methods for DST were conventional methods on solid media, these methods are slow and take a minimum of 3 to 8 weeks before results are available [8,9]. Susceptibility results had to be reported within 4 weeks of specimen receipt [10]. The WHO recommends the use of liquid media for culture and DST in middle- and low-income countries to improve diagnosis of MDR- and XDR-TB [11,12]. The BACTEC MGIT 960 system (Becton Dickinson Microbiology System, Sparks, NV, USA) is currently regarded as an excellent method able to provide rapid and reliable results of susceptibility testing [11,12]. The BACTEC MGIT 960 system is an automated, continuously monitoring system, based on the detection of bacterial growth in drug-containing media which is compared with a drug-free control tube [13]. Drug susceptibility kits are available for the testing of INH, RIF, EMB, STR and PZA [13]. The BACTEC MGIT 960 system has been evaluated for the detection of resistance to first-line anti-TB drugs against the standard proportion method and the BACTEC 460 TB system and has shown a sensitivity of 100% for RIF and INH and a specificity ranging from 89% to 100% [14-17].

The BACTEC MGIT 960 system was also evaluated for DST of second-line anti-TB drugs and critical concentrations for second-line drugs has been established [18-21]. The BACTEC MGIT 960 system has shown good concordance with the standard proportion method [15-18]. Currently, there is no commercially available kit for performing DST of second-line anti-TB drugs using the BACTEC MGIT 960 system [20,21]. This is inconvenient because the working solutions of each drug should be prepared by the users, making these tests more error-prone due to procedural inaccuracies. Testing for second-line anti-TB drugs should be precise and quality controlled using the reference *M. tuberculosis* H37Rv strain and resistant *M. tuberculosis* strains.

The aim of the study was to determine the reliability and accuracy of the routine susceptibility testing of EMB, STR, KAN and OFX at a high-throughput diagnostic laboratory in a high-burden setting. The susceptibility results of MDR-TB isolates using the BACTEC MGIT 960 system (Becton Dickinson, Sparks, Md, USA) were compared against the agar proportion method.

## Results

Routine DST results for STR and EMB using the BACTEC MGIT 960 system were available for all the MDR-TB isolates, however, only 205 MDR-TB isolates; had results for second-line anti-TB drugs (OFX and KAN). The results of the routine DST using the BACTEC MGIT 960 system were compared to the results obtained by the agar proportion method. The results are summarized in Table 1. The mean time to obtain susceptibility testing results for resistant isolates with the BACTEC MGIT 960 system was 6 days, with a range of 5 to 14 days whereas the agar proportion method was 11 days, with a range of 7 to 21 days.

**Table 1:** Susceptibility testing results obtained with the BACTEC MGIT 960 system and the agar proportion method

**Drug**	**No of isolates**	**Both S**	**Both R**	**MGIT 960 R, proportion S**	**MGIT 960 S, proportion R**	**Agreement (%)**
STR	343	74	138	124	7	61
EMB	343	110	43	186	4	44
OFX	205	171	5	1	22	89
KAN	205	178	6	5	16	89

*S* = Susceptible; *R* = Resistant, *STR* = Streptomycin, *EMB* = Ethambutol, *OFX* = Ofloxacin, *KAN* = Kanamycin.

The prevalence of resistance using the BACTEC MGIT 960 system and the agar proportion method was as follows: 76% (262/343) and 42% (145/343) for STR, 67% (229/343) and 14% (47/343) for EMB, 2% (5/205) and 13% (27/205) for OFX and 5% (11/205) and 11% (22/205) for KAN, respectively. A total of 3% (6/205) MDR-TB isolates met the criteria for classification as XDR-TB according to the BACTEC MGIT 960 system and 7% (15/205) according to the agar proportion method.

The agreement between the BACTEC MGIT 960 system and the agar proportion method was 61% for STR and 44% for EMB and 89% for OFX as well as KAN (Table 1). The κ values are shown in Table 2. The sensitivity and specificity of the BACTEC MGIT 960 system was 95% and 37% for STR, 91% and 37% for EMB, 84% and 90% for OFX, 27% and 97% for KAN, respectively (Table 2).

**Table 2:** Performance of the BACTEC MGIT 960 system when compared to the agar proportion method

**Drug**	**No of isolates**	**Sensitivity (%)**	**Specificity (%)**	**PPV (%)**	**NPV (%)**	**κ -Value**
STR	343	95	37	53	91	0.29
EMB	343	92	37	18	96	0.11
OFX	205	84	90	18	99	0.282
KAN	205	27	97	55	92	0.314

*STR* = Streptomycin, *EMB* = Ethambutol, *OFX* = Ofloxacin, *KAN* = Kanamycin.

## Discussion

The study describes the performance of BACTEC MGIT 960 system for testing of MDR-TB isolates against EMB, STR, KAN and OFX in a routine diagnostic laboratory. According to WHO recommendations, additional testing for the remaining first-line anti-TB drugs and second-line anti-TB drugs should be done once MDR-TB has been confirmed [22]. Reliable and accurate susceptibility testing of first and second-line anti-TB drugs is essential in order to determine an effective treatment regimen of MDR-TB and to avoid further development of resistance [1,23]. The accuracy of susceptibility testing results varies with the drug tested as well as with the method of drug susceptibility testing used. In this study the performance of the BACTEC MGIT 960 system for routine DST of MDR-TB isolates was compared against the agar proportion method using Middlebrook medium.

The BACTEC MGIT 960 system performed well for STR and EMB when compared to the agar proportion method (sensitivity in detecting resistance, 95% and 92%; respectively); although the specificity was low for both drugs noting that we had a large number of strains tested. This finding could be due to a number of factors. It is known that specificity can be affected by technical errors, such as transfer of TB-bacilli from positive to negative samples. It should be remembered that the BACTEC MGIT 960 system was done in a high throughput routine setting, while the proportion method was done in a research facility. Differences in the medium composition or technical differences in the procedure used for growth measurement between the two methods could also affect specificity. In addition, the MIC distributions can vary depending on the susceptibility testing method used as well as the geographic setting and the prevalence of these strains. It has been reported that the susceptibility of *M. tuberculosis* to EMB, STR and PZA is less reliable and reproducible using solid medium [24-26]. Among the supranational reference laboratories of the WHO, the accepted minimum performance level (the proportion of concordant results) for the testing of susceptibility to STR and EMB is only 92% (26). Especially the susceptibility testing of EMB has long been a challenge for diagnostic laboratories. Discrepancies have been reported when comparing results obtained in liquid versus solid media [26-29].

The performance of BACTEC MGIT 960 system for OFX (84% sensitivity and 90% specificity) was within the range to that reported previously by Devasia et al. [30], where *M. tuberculosis* isolates from culture-confirmed TB patients from 2002 to 2007 were tested for OFX resistance by proportion method and BACTEC MGIT 960. The reported sensitivity for OFX was 83% to 100%, although the specificity was higher (99.5% to 100%) when compared to this study. The sensitivity in detecting resistance to KAN were considerably lower (27%), whereas the specificity was excellent (97%). Previous studies reported higher sensitivity values (90% to 100%) for KAN [31,32]. This could be due the poor standardisation of second-line anti-TB drug testing.

The values of positive predictive value (PPV) and negative predictive value (NPV) vary markedly with the prevalence of the disease in a given community. In this study, the PPV for STR, EMB and OFX were low, indicating the BACTEC MGIT 960 system could not be used in the study setting. However, the PPV of a test is primarily affected by the specificity; i.e., the lower the specificity the lower the PPV of a test will be. The PPV in this study may be further decreased due to the nature of the study population. In this study only MDR-TB strains were used which changes the pre-test probability. The strain difference may also affect test performance. In high-burden TB courtiers such as South Africa, sensitivity needs to be high to reach a good NPV. Countries with low prevalence, specificity needs to be very high; otherwise the PPV of a test will be poor.

In this study the performance of the BACTEC MGIT 960 system were generally lower than those obtained by other investigators. Previous evaluation studies for MGIT 960 system for testing first-line anti-TB drugs including multicenter studies reported excellent performance especially for RIF and INH [14-16,18]. Excellent agreement was also reported for second-line anti-TB against the proportion method [33,34] or broth-based methods such as colometric methods [35]. The MGIT 960 system is currently recommended by the WHO as the gold standard for second-line DST [36]. In case of first-line anti-TB drugs, the WHO recommends the use of both the BACTEC MGIT 960 system and the line-probe assay for testing [37]. However, confirmation of MDR-TB by conventional solid-based DST is still regarded as the gold standard for first-line anti-TB drugs [37].

The lower performance of MGIT 960 in this study when compared to the agar proportion method could be due to the presence of borderline resistant *M. tuberculosis* strains, mainly in relation to the agar proportion method where the final results depend on an accurate count of colonies [22]. In addition, the critical drug concentration defining resistance for EMB and second-line drugs is often very close to the MIC required to achieve antimycobacterial activity, increasing the probability of misclassification of susceptibility or resistance, and leading to poor reproducibility of DST results. The discordance between the BACTEC MGIT 960 system and agar proportion method might possibly be overcome by adjusting the critical drug concentrations used. In addition, the use of two concentrations (low and high) for these drugs may reduce false resistance.

A limitation of the study was the lack of resolution of the discrepant results between the BACTEC MGIT 960 system and the agar proportion using DNA sequencing. However, this study provides a routine testing scenario where sequencing is rarely available.

## Conclusion

The BACTEC MGIT 960 system showed acceptable sensitivity for EMB, STR, and OFX; however, the BACTEC MGIT 960 system was less specific for EMB and STR and demonstrated a low sensitivity for KAN. The lower agreement found between the two methods suggests the unreliability of the BACTEC MGIT 960 system for the drugs tested. The reasons for the lower agreement between the two methods need to be investigated and further studies are needed in this setting to confirm the study finding.

## Methods

### Study design

This study was a descriptive study comparing the performance of the BACTEC MGIT 960 system with the standard agar proportion method for susceptibility of first and second-line anti-TB drugs. The diagnostic performance such as: sensitivity, specificity and predictive values were calculated using the agar proportion method as gold standard.

### Specimens

The study was conducted using consecutive *M. tuberculosis* isolates identified as MDR-TB during August 2007 and January 2008 by routine DST using the BACTEC MGIT 960 system (Becton Dickinson, Sparks, Md, USA) at the National Health Laboratory Service (NHLS) laboratory, University of Limpopo (MEDUNSA campus). The NHLS laboratory is a high-throughput routine diagnostic microbiology laboratory that receives specimens for culture and DST from the attached Dr George Mukhari Hospital and the surrounding clinics and hospitals in Gauteng as well as the referring provinces of Limpopo, Mpumalanga and North-West. All the MDR-TB isolates were tested by the agar proportion method.

### Routine culture and drug susceptibility testing

Routine drug susceptibility testing for EMB, STR, KAN and OFX was done by NHLS laboratory using the BACTEC MGIT 960 system. Susceptibility testing for EMB and STR was done as part of the MGIT AST SIRE kit (Becton Dickinson, Sparks, Md., USA) as described in the MGIT DST package insert [13]. For KAN and OFX, the standard operating protocol for DST of second-line anti-TB drugs from the NHLS was followed, with critical concentrations showed in Table 3. The drug susceptibility testing results were collected from the NHLS data-base. Isolation of the recovered mycobacterial isolates from the clinical specimens was performed using the BACTEC MGIT 960 system. The isolates were identified as *M. tuberculosis* using Ziehl-Neelsen (ZN) staining and were confirmed with the Accuprobe method (Gen-Probe, Inc, San Diego, California).

**Table 3:** Drug concentrations used for the BACTEC MGIT 960 system and the agar proportion method

**Drug**	**BACTEC MGIT 960 system (μg/ml)**	**Agar proportion method (μg/ml)**
STR	1	2
EMB	5	7.5
OFX	1	2
KAN	5	5

*STR* = Streptomycin, *EMB* = Ethambutol, *OFX* = Ofloxacin, *KAN* = Kanamycin.

### Drug susceptibility testing using the agar proportion method

All MDR-TB isolates were sub-cultured on Middlebrook medium prior to testing. Drug susceptibility testing of MDR-TB isolates using the agar proportion method was done for two first-line (EMB and STR) and two second-line drugs (KAN and OFX). The agar proportion method was performed on Middlebrook 7H11 medium according to the Clinical and Laboratory Standards Institute (CLSI) procedures and recommended critical concentrations (Table 3) [38]. Briefly, six-welled Petri plates of Middlebrook7H11 medium (TB Diagnostic Services, South Africa) was used. Two quadrants in each plate contained drug-free medium, one was used as the proportional control and the other was used as a quality control. Fully susceptible *M. tuberculosis* H37Rv reference strain and a known MDR *M. tuberculosis* isolate were used as quality controls. The other four quadrants contained the drug concentrations (Table 3). Each quadrant was inoculated with a standard inoculum of 0.1 ml of mycobacterial suspension and the inoculum was distributed by tilting the plate. An aliquot of 0.1 ml of the 1:100 dilutions was used to inoculate the proportional control. The plates were sealed in a plastic bag and incubated at 37 °C. The plates were examined 7, 10, 14 and 21 days of incubation. An isolate was classified as resistant when the colonies on the drug-containing quadrant were more than 1% compared to the colonies present on the drug-free control quadrant. An XDR-TB was defined as MDR-TB with additional resistance to KAN and OFX.

### Analysis

The results were expressed as percentages. The agreement, sensitivity, specificity, positive and negative predictive values of the BACTEC MGIT 960 system compared to the agar proportion method, the gold standard, were calculated for EMB, STR, KAN and OFX. The agreement between the two methods was determined by the kappa (κ) statistic. The κ value, a measure of test reliability, was interpreted as follows: < 0.2, poor; 0.21 to 0.4, fair; 0.41 to 0.6, moderate; 0.61 to 0.8, good; ≥ 0.81, excellent [39].

### Ethical

Approval for the study protocol (MCREC/P/07/2008) was obtained from the Ethics Committee of the Faculty of Health Sciences, University of Pretoria and University of Limpopo.

## Competing interests

The authors declare that they have no competing interests.

## Authors’ contributions

All the authors planned and designed the study. HM Said was responsible for all the practical laboratory work, data collection, analysis and interpretation of the data and drafted the manuscript. MM Kock, NA Ismail, K Baba, SV Omar, A Osman, AA Hoosen and MM Ehlers critically reviewed the manuscript versions. All authors read and approved the final manuscript.

## Pre-publication history

The pre-publication history for this paper can be accessed here:

http://www.biomedcentral.com/1471-2334/12/369/prepub

## References

[B1] World Health Organization Multidrug and extensively drug-resistant TB (M/XDR-TB) 2010 Global report on surveillance and response 2010 Geneva, Switzerland: World Health Organization

[B2] Centers for Disease Control and Prevention Notice to readers: Revised definition of extensively drug-resistant tuberculosis MMWR 2006 55 1176

[B3] Palomino JC Martin A Von Groll A Portaels F Rapid culture-based methods for drug-resistance detection in *Mycobacterium* tuberculosis J Microbiol Methods 2008 75 161 166 10.1016/j.mimet.2008.06.015 18627779

[B4] Richter E Rüsch-Gerdes S Hillemann D Drug-susceptibility testing in TB: current status and future prospects Expert Rev Respir Med 2009 3 497 510 10.1586/ers.09.45 20477339

[B5] O’Grady J Maeurer M Mwaba P Kapata N Bates M Hoelscher M Zumla A New and improved diagnostics for detection of drug resistant pulmonary tuberculosis Curr Opin Pulm Med 2011 17 134 141 10.1097/MCP.0b013e3283452346 21415753

[B6] Parsons LM Somoskövi A Urbanczik R Salfinger M Laboratory diagnostic aspects of drug resistant tuberculosis Front Biosci 2004 9 2086 2105 10.2741/1290 15353272

[B7] Johnson R Jordaan AM Pretorius L Engelke E van der Spuy G Kewley C Bosman M Van Helden PD Warren R Victor TC Ethambutol resistance testing by mutation detection Int J Tuberc Lung Dis 2006 10 68 73 16466040

[B8] Canetti G Froman F Grosset J Hauduroy P Langerova M Mahler HT Meissner G Mitchison DA Sula L Mycobacteria: laboratory methods for testing drug sensitivity and resistance Bull WHO 1963 29 565 578 14102034 PMC2555065

[B9] Heifets LB Drug susceptibility in chemotherapy of mycobacterial infections 2000 1 Florida: CRC press

[B10] Tenover FC Crawford JT Huebner RE Geiter LJ Horsburgh CR Jr Good RC The resurgence of tuberculosis: is your laboratory ready? J Clin Microbiol 1993 31 767 770 8463384 10.1128/jcm.31.4.767-770.1993 PMC263557

[B11] Parrish N Carrol K Importance of improved TB diagnostics in addressing the extensively drug-resistant TB crisis Future Microbiol 2008 3 405 413 10.2217/17460913.3.4.405 18651812

[B12] World Health Organization Strategic and technical advisory group for tuberculosis (STAG-TB). Report on conclusions and recommendations11 to 13 June 2007 2007 Geneva, Switzerland: World Health Organization

[B13] BD BACTEC™ MGIT™ 960 SIRE kit for the antimycobacterial susceptibility testing of Mycobacterium tuberculosis 2002 Franklin Lakes, NJ, USA: Becton Dickinson and Company

[B14] Rüsch-Gerdes S Domehl C Nardi G Gismondo MR Welscher HM Pfyffer GE Multicenter evaluation of the mycobacteria growth indicator tube for testing susceptibility of *Mycobacterium tuberculosis* to first-line drugs J Clin Microbiol 1999 37 45 48 9854062 10.1128/jcm.37.1.45-48.1999 PMC84164

[B15] Ardito F Posteraro B Sanguinetti M Zanetti S Fadda G Evaluation of BACTEC Mycobacteria Growth Indicator Tube (MGIT 960) automated system for drug susceptibility testing of *Mycobacterium tuberculosis* J Clin Microbiol 2001 39 4440 4444 10.1128/JCM.39.12.4440-4444.2001 11724858 PMC88562

[B16] Bemer P Palicova F Rusch-Gerdes S Drugeon HB Pfyffer GE Multicenter evaluation of fully automated BACTEC Mycobacteria Growth Indicator Tube 960 system for susceptibility testing of *Mycobacterium tuberculosis* J Clin Microbiol 2002 40 150 154 10.1128/JCM.40.1.150-154.2002 11773109 PMC120114

[B17] Tortoli E Benedetti M Fontanelli A Simonetti MT Evaluation of automated BACTEC MGIT 960 system for testing susceptibility of *Mycobacterium tuberculosis* to four major anti-tuberculous drugs: comparison with the radiometric BACTEC 460TB method and the agar plate method of proportion J Clin Microbiol 2002 40 607 610 10.1128/JCM.40.2.607-610.2002 11825978 PMC153389

[B18] Krüüner A Yates MD Drobniewski FA Evaluation of MGIT 960-based antimicrobial testing and determination of critical concentrations of first- and second-line antimicrobial drugs with drug-resistant clinical strains of *Mycobacterium tuberculosis* J Clin Microbiol 2006 44 811 818 10.1128/JCM.44.3.811-818.2006 16517859 PMC1393078

[B19] Rüsch-Gerdes S Pfyffer GE Casal M Chadwick M Siddiqi S Multicenter laboratory validation of the BACTEC MGIT 960 technique for testing susceptibilities of *Mycobacterium tuberculosis* to classical second-line drugs and newer antimicrobials J Clin Microbiol 2006 44 688 692 10.1128/JCM.44.3.688-692.2006 16517840 PMC1393114

[B20] Rodrigues C Jani J Shenai S Thakkar P Siddiqi S Mehta A Drug susceptibility testing of *Mycobacterium tuberculosis* against second-line drugs using the BACTEC MGIT 960 System Int J Tuberc Lung Dis 2008 12 1449 1455 19017456

[B21] Lin SY Desmond E Bonato D Gross W Siddiqi S Multicenter evaluation of BACTEC MGIT 960 system for second-line drug susceptibility testing of *Mycobacterium tuberculosis* complex J Clin Microbiol 2009 47 3630 3634 10.1128/JCM.00803-09 19741086 PMC2772628

[B22] World Health Organization Policy guidance on drug susceptibility testing (DST) of second-line anti-tuberculosis drugs WHO/HTM/TB/2008.392 2008 Geneva, Switzerland: World Health Organization 26290924

[B23] World Health Organization Guidelines for drug susceptibility testing for second line anti-tuberculosis drugs for DOTS-Plus Document no. WHO/CDS/TB/2001.288 2001 Geneva, Switzerland: World Health Organization

[B24] World Health Organization The WHO/IUATLD global project on anti-tuberculosis drug resistance surveillance Anti-tuberculosis drug resistance in the world, report no. 2: prevalence and trends 2000 Geneva, Switzerland: World Health Organization

[B25] Laszlo A Rahman M Raviglione M Bustreo F Quality assurance programme for drug susceptibility testing of *Mycobacterium tuberculosis* in the WHO/IUATLD Supranational Laboratory Network: first round of proficiency testing Int J Tuberc Lung Dis 1997 1 231 238 9432369

[B26] Laszlo A Rahman M Espinal M Raviglione M WHO/IUATLD Network of Supranational Reference Laboratories: Quality assurance programme for drug susceptibility testing of *Mycobacterium tuberculosis* in the WHO/IUATLD Supranational Reference Laboratory Network: five rounds of proficiency testing, 1994–1998 Int J Tuberc Lung Dis 2002 6 748 756 12234129

[B27] Woodley CL Evaluation of streptomycin and ethambutol concentrations for susceptibility testing of *Mycobacterium tuberculosis* by radiometric and conventional procedures J Clin Microbiol 1986 23 385 386 3084552 10.1128/jcm.23.2.385-386.1986 PMC268651

[B28] Van Rie A Warren R Mshanga I Jordaan AM Van der Spuy GD Richardson M Simpson J Gie RP Enarson DA Beyers N Van Helden PD Victor TC Analysis for a limited number of gene codons can predict drug-resistance of *Mycobacterium tuberculosis* in a high-incidence community J Clin Microbiol 2001 39 636 641 10.1128/JCM.39.2.636-641.2001 11158121 PMC87790

[B29] Mokrousov I Narvskaya O Limeschenko E Otten T Vyshnevskiy B Detection of ethambutol-resistant *Mycobacterium tuberculosis* strains by multiplex allele-specific PCR assay targeting embB306 mutations J Clin Microbiol 2002 40 1617 1620 10.1128/JCM.40.5.1617-1620.2002 11980930 PMC130919

[B30] Devasia RA Blackman A May C Eden S Smith T Hooper N Maruri F Stratton S Shintani A Sterling TR Fluoroquinolone resistance in*Mycobacterium tuberculosis*: an assessment of MGIT 960, MODS and nitrate reductase assay and fluoroquinolone cross-resistance. J Antimicrob Chemother 2009 63 1173 1178 10.1093/jac/dkp096 19329799 PMC2680343

[B31] Martin A Von Groll A Fissette K Palomino JC Varaine F Portaels F Rapid detection of *Mycobacterium tuberculosis* resistance to second-line drugs by use of the manual Mycobacterium growth indicator tube system J Clin Microbiol 2008 46 3952 3956 10.1128/JCM.01171-08 18945838 PMC2593252

[B32] Bastian I Rigouts L Palomino JC Portaels F Kanamycin Susceptibility Testing of *Mycobacterium tuberculosis* using Mycobacterium Growth Indicator Tube and a Colorimetric method Antimicrob Agents Chemother 2001 45 1934 1936 10.1128/AAC.45.6.1934-1936.2001 11353658 PMC90578

[B33] Juréen P Ängeby K Sturegård E Chryssanthou E Giske CG Werngren J Nordvall M Johansson A Kahlmeter G Hoffner S Schön T Wild-Type MIC distributions for aminoglycoside and cyclic polypeptide antibiotics used for treatment of *Mycobacterium tuberculosis* Infections J Clin Microbiol 2010 48 1853 1858 10.1128/JCM.00240-10 20237102 PMC2863855

[B34] Ängeby K Giske CG Juréen P Schön T Wild-Type MIC distributions must be considered to set clinically meaningful susceptibility testing breakpoints for all bacterial pathogens, including *Mycobacterium tuberculosis* Antimicrob Agents Chemother 2011 55 4492 4493 10.1128/AAC.00232-11 21849570 PMC3165346

[B35] Morcillo N Imperiale B Di Giulio B Evaluation of MGIT 960 and the colorimetric-based method for tuberculosis drug susceptibility testing Int J Tuberc Lung Dis 2010 14 1169 1175 20819264

[B36] World Health Organization Framework for implementing new tuberculosis diagnostics 2010 Geneva, Switzerland: World Health Organization

[B37] World Health Organization Molecular line probe assays for rapid screening of patients at risk of multidrug resistant tuberculosis 2008 Geneva, Switzerland: World Health Organization

[B38] National Committee for Clinical Laboratory Standards Susceptibility testing for Mycobacteria, Nocardiae, and other aerobic Actinomycetes; approved standard. Volume 23: (18), NCCLS document, pM-24A 2003 NCCLS: Wayne, PA 31339680

[B39] Landis JR Koch GG The measurement of observer agreement for categorical data In Biometrics 1977 Volume 33 159 174 10.2307/2529310 843571

